# 1,4-Diazo­niabicyclo­[2.2.2]octane diaqua­dichlorido­(oxalato-κ^2^ *O*,*O*′)iron(III) chloride

**DOI:** 10.1107/S1600536809025628

**Published:** 2009-07-08

**Authors:** Ying Cai

**Affiliations:** a Ordered Matter Science Research Center, Southeast University, Nanjing 211189, People’s Republic of China

## Abstract

In the title compound, (C_6_H_14_N_2_)[Fe(C_2_O_4_)Cl_2_(H_2_O)_2_]Cl, all ions are situated on twofold rotational axes. The Fe^III^ ion is coordinated by two O atoms from a chelating oxalate ligand, two water mol­ecules and two chloride anions in a distorted octa­hedral geometry. Inter­molecular N—H⋯O, O—H⋯O and O—H⋯Cl hydrogen bonds form an extensive three-dimensional network which consolidates the crystal packing.

## Related literature

For the crystal structures of related compounds, see: Fu *et al.* (2002 ▶); Keene *et al.* (2004 ▶); Sukhendu & Srinivasan (2007 ▶); Zhao & Xu (2008 ▶); Lee & Wang (1999 ▶).
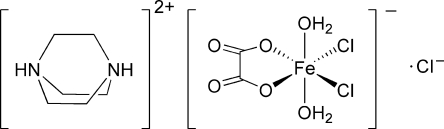


## Experimental

### 

#### Crystal data

(C_6_H_14_N_2_)[Fe(C_2_O_4_)Cl_2_(H_2_O)_2_]Cl
*M* *_r_* = 400.44
Monoclinic, 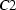

*a* = 9.872 (2) Å
*b* = 9.6636 (19) Å
*c* = 8.4268 (17) Å
β = 109.57 (3)°
*V* = 757.4 (3) Å^3^
*Z* = 2
Mo *K*α radiation
μ = 1.55 mm^−1^
*T* = 293 K
0.30 × 0.30 × 0.20 mm

#### Data collection

Rigaku Mercury CCD diffractometer
Absorption correction: multi-scan (*CrystalClear*; Rigaku, 2005 ▶) *T*_min_ = 0.638, *T*_max_ = 0.734
3954 measured reflections
1729 independent reflections
1684 reflections with *I* > 2σ(*I*)
*R*_int_ = 0.025

#### Refinement

*R*[*F* ^2^ > 2σ(*F* ^2^)] = 0.020
*wR*(*F* ^2^) = 0.049
*S* = 1.08
1729 reflections
101 parameters
1 restraint
H atoms treated by a mixture of independent and constrained refinement
Δρ_max_ = 0.17 e Å^−3^
Δρ_min_ = −0.15 e Å^−3^
Absolute structure: Flack (1983 ▶), 802 Friedel pairs
Flack parameter: 0.016 (13)

### 

Data collection: *CrystalClear* (Rigaku, 2005 ▶); cell refinement: *CrystalClear*; data reduction: *CrystalClear*; program(s) used to solve structure: *SHELXS97* (Sheldrick, 2008 ▶); program(s) used to refine structure: *SHELXL97* (Sheldrick, 2008 ▶); molecular graphics: *SHELXTL*/*PC* (Sheldrick, 2008 ▶); software used to prepare material for publication: *SHELXL97*.

## Supplementary Material

Crystal structure: contains datablocks I, global. DOI: 10.1107/S1600536809025628/cv2580sup1.cif

Structure factors: contains datablocks I. DOI: 10.1107/S1600536809025628/cv2580Isup2.hkl

Additional supplementary materials:  crystallographic information; 3D view; checkCIF report

## Figures and Tables

**Table 1:** Hydrogen-bond geometry (Å, °)

*D*—H⋯*A*	*D*—H	H⋯*A*	*D*⋯*A*	*D*—H⋯*A*
N1—H1⋯O1^i^	0.91	1.93	2.814 (2)	162
O2—H2*WA*⋯O3^ii^	0.87 (3)	1.86 (3)	2.722 (2)	168 (2)
O2—H2*WB*⋯Cl2	0.82 (3)	2.23 (3)	3.0359 (17)	170 (3)

Symmetry codes: (i) 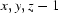; (ii) 
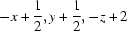
.

**Fig. 1.**
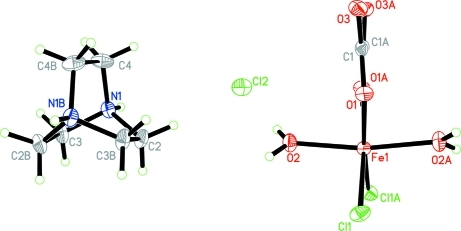
A view of (1) with the atomic numbering scheme. Displacement ellipsoids were drawn at the 30% probability level. Symmetry codes: (A) -x, y, -z + 2; (B) -x + 1, y, -z + 1.

**Fig. 2.**
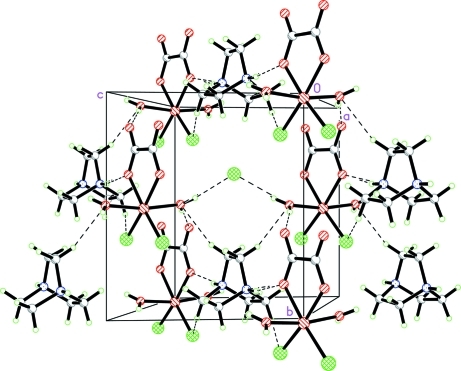
The crystal packing viewed along the a axis. Hydrogen atoms not involved in hydrogen bonding (dashed lines) were omitted for clarity.

## supplementary crystallographic information

### Comment

Oxalic acid is often used as bridging ligand, which can adopt different coordination modes according to the different geometric requirements of metal centers when forming metal complexes (Sukhendu & Srinivasan, 2007; Zhao & Xu, 2008). We report here the crystal structure of the title compound, (1).

The stucture of (1) is shown in Fig. 1. This yellow ionic compound crystallizes in the monoclinic space group C2. It contains [Fe(ox)(H_2_O)_2_Cl_2_]^-^ (ox is oxalate, C_2_O_4_) units, in which the Fe^III^ ion is coordinated by two O atoms from a chelating oxalato ion, two O atoms from coordinated water molecules and two Cl anions, forming a distorted octahedron coordination geometry. The crystal packing is stabilized by  N—H···O, O—H···O and O—H···Cl hydrogen bonds (Table 1, Fig. 2).

### Experimental

A mixture of oxalic acid (0.01 mol 0.9 g) and iron(III) chloride (0.01 mol 1.62 g) and the 1,4-diaza-bicyclo[2.2.2]octane (dabco) (0.01 mol 1.12 g) in H_2_O (20 ml) was stirred until clear. Adjust the pH value of the solution to 4 with 10% HCl solution. After slow evaporation, yellow plate crystals of the title compand suitable for X-ray analysis were obtained with about 65% yield (based on Fe).

### Refinement

H atoms bound to C and N atoms were positioned geometrically and refined as riding, with C—H = 0.97 and N—H = 0.91 Å, and with *U*_iso_(H) = 1.2*U*_eq_(parent atom). H atoms bound to O atoms were located in difference maps, but their  O—H distances and H—O—H angles were restrained to the literature values.

### Crystal data

**Table:**

(C_6_H_14_N_2_)[Fe(C_2_O_4_)Cl_2_(H_2_O)_2_]Cl	*F*(000) = 410
*M**_r_* = 400.44	*D*_x_ = 1.756 Mg m^−^^3^
Monoclinic, *C*2	Mo *K*α radiation, λ = 0.71073 Å
Hall symbol: C 2y	Cell parameters from 3950 reflections
*a* = 9.872 (2) Å	θ = 3.0–27.5°
*b* = 9.6636 (19) Å	µ = 1.55 mm^−^^1^
*c* = 8.4268 (17) Å	*T* = 293 K
β = 109.57 (3)°	Plate, yellow
*V* = 757.4 (3) Å^3^	0.30 × 0.30 × 0.20 mm
*Z* = 2	

### Data collection

**Table:**

Rigaku Mercury CCD diffractometer	1729 independent reflections
Radiation source: fine-focus sealed tube	1684 reflections with *I* > 2σ(*I*)
graphite	*R*_int_ = 0.025
ω scans	θ_max_ = 27.5°, θ_min_ = 3.0°
Absorption correction: multi-scan (*CrystalClear*; Rigaku, 2005)	*h* = −12→12
*T*_min_ = 0.638, *T*_max_ = 0.734	*k* = −12→12
3954 measured reflections	*l* = −10→10

### Refinement

**Table:**

Refinement on *F*^2^	Hydrogen site location: inferred from neighbouring sites
Least-squares matrix: full	H atoms treated by a mixture of independent and constrained refinement
*R*[*F*^2^ > 2σ(*F*^2^)] = 0.020	*w* = 1/[σ^2^(*F*_o_^2^) + (0.017*P*)^2^] where *P* = (*F*_o_^2^ + 2*F*_c_^2^)/3
*wR*(*F*^2^) = 0.049	(Δ/σ)_max_ < 0.001
*S* = 1.08	Δρ_max_ = 0.17 e Å^−^^3^
1729 reflections	Δρ_min_ = −0.15 e Å^−^^3^
101 parameters	Extinction correction: *SHELXL97* (Sheldrick, 2008)
1 restraint	Extinction coefficient: 0.0476 (15)
Primary atom site location: structure-invariant direct methods	Absolute structure: Flack (1983), 802 Friedel pairs
Secondary atom site location: difference Fourier map	Flack parameter: 0.016 (13)

### Special details

**Table:**

Geometry. All e.s.d.'s (except the e.s.d. in the dihedral angle between two l.s. planes) are estimated using the full covariance matrix. The cell e.s.d.'s are taken into account individually in the estimation of e.s.d.'s in distances, angles and torsion angles; correlations between e.s.d.'s in cell parameters are only used when they are defined by crystal symmetry. An approximate (isotropic) treatment of cell e.s.d.'s is used for estimating e.s.d.'s involving l.s. planes.
Refinement. Refinement of *F*^2^ against ALL reflections. The weighted *R*-factor *wR* and goodness of fit *S* are based on *F*^2^, conventional *R*-factors *R* are based on *F*, with *F* set to zero for negative *F*^2^. The threshold expression of *F*^2^ > σ(*F*^2^) is used only for calculating *R*-factors(gt) *etc*. and is not relevant to the choice of reflections for refinement. *R*-factors based on *F*^2^ are statistically about twice as large as those based on *F*, and *R*- factors based on ALL data will be even larger.

### Fractional atomic coordinates and isotropic or equivalent isotropic displacement parameters (Å^2^)

**Table:**

	*x*	*y*	*z*	*U*_iso_*/*U*_eq_	
Fe1	0.0000	1.02078 (3)	1.0000	0.02213 (11)	
Cl1	0.16532 (6)	1.17560 (5)	1.16036 (7)	0.03727 (16)	
Cl2	0.0000	0.85379 (10)	0.5000	0.0462 (2)	
N1	0.37619 (17)	0.90236 (18)	0.3965 (2)	0.0285 (4)	
H1	0.2853	0.9021	0.3207	0.034*	
C1	0.07314 (19)	0.7358 (2)	1.0751 (2)	0.0242 (4)	
C2	0.3777 (2)	0.9825 (2)	0.5494 (2)	0.0377 (5)	
H2A	0.3047	0.9470	0.5922	0.045*	
H2B	0.3572	1.0792	0.5207	0.045*	
C3	0.4750 (2)	0.9679 (3)	0.3179 (3)	0.0393 (5)	
H3A	0.4386	1.0582	0.2735	0.047*	
H3B	0.4812	0.9111	0.2257	0.047*	
C4	0.4218 (3)	0.7572 (2)	0.4454 (3)	0.0464 (6)	
H4A	0.4088	0.7017	0.3454	0.056*	
H4B	0.3638	0.7176	0.5067	0.056*	
O1	0.11725 (13)	0.85657 (14)	1.13039 (16)	0.0273 (3)	
O2	0.11176 (16)	1.00290 (19)	0.83598 (18)	0.0354 (4)	
O3	0.13212 (14)	0.62670 (16)	1.1287 (2)	0.0375 (4)	
H2WA	0.199 (3)	1.033 (3)	0.859 (3)	0.046 (7)*	
H2WB	0.075 (3)	0.971 (4)	0.741 (4)	0.075 (10)*	

### Atomic displacement parameters (Å^2^)

**Table:**

	*U*^11^	*U*^22^	*U*^33^	*U*^12^	*U*^13^	*U*^23^
Fe1	0.01824 (19)	0.0248 (2)	0.01995 (18)	0.000	0.00185 (14)	0.000
Cl1	0.0293 (3)	0.0348 (3)	0.0404 (3)	−0.0041 (2)	0.0021 (2)	−0.0136 (2)
Cl2	0.0591 (5)	0.0447 (5)	0.0256 (4)	0.000	0.0019 (3)	0.000
N1	0.0172 (8)	0.0399 (10)	0.0226 (8)	−0.0024 (7)	−0.0010 (7)	0.0007 (7)
C1	0.0175 (9)	0.0296 (10)	0.0273 (9)	0.0010 (7)	0.0099 (8)	0.0025 (7)
C2	0.0249 (10)	0.0499 (15)	0.0397 (11)	0.0031 (9)	0.0125 (9)	−0.0086 (10)
C3	0.0269 (10)	0.0608 (14)	0.0304 (10)	0.0065 (9)	0.0098 (9)	0.0190 (10)
C4	0.0521 (15)	0.0290 (13)	0.0477 (15)	−0.0115 (11)	0.0030 (13)	−0.0061 (11)
O1	0.0207 (7)	0.0276 (7)	0.0262 (7)	0.0018 (6)	−0.0019 (5)	0.0003 (6)
O2	0.0291 (8)	0.0488 (10)	0.0300 (7)	−0.0128 (7)	0.0122 (6)	−0.0117 (8)
O3	0.0294 (7)	0.0313 (8)	0.0520 (10)	0.0096 (6)	0.0139 (7)	0.0145 (7)

### Geometric parameters (Å, °)

**Table:**

Fe1—O2^i^	2.0443 (14)	C1—C1^i^	1.569 (4)
Fe1—O2	2.0443 (14)	C2—C3^ii^	1.515 (3)
Fe1—O1^i^	2.0526 (14)	C2—H2A	0.9700
Fe1—O1	2.0526 (14)	C2—H2B	0.9700
Fe1—Cl1	2.2913 (8)	C3—C2^ii^	1.515 (3)
Fe1—Cl1^i^	2.2913 (8)	C3—H3A	0.9700
N1—C4	1.489 (2)	C3—H3B	0.9700
N1—C3	1.491 (3)	C4—C4^ii^	1.509 (5)
N1—C2	1.499 (2)	C4—H4A	0.9700
N1—H1	0.9100	C4—H4B	0.9700
C1—O3	1.216 (2)	O2—H2WA	0.87 (3)
C1—O1	1.278 (2)	O2—H2WB	0.82 (3)
			
O2^i^—Fe1—O2	170.30 (10)	O1—C1—C1^i^	113.78 (10)
O2^i^—Fe1—O1^i^	87.78 (6)	N1—C2—C3^ii^	108.47 (16)
O2—Fe1—O1^i^	84.72 (6)	N1—C2—H2A	110.0
O2^i^—Fe1—O1	84.72 (6)	C3^ii^—C2—H2A	110.0
O2—Fe1—O1	87.78 (6)	N1—C2—H2B	110.0
O1^i^—Fe1—O1	78.73 (8)	C3^ii^—C2—H2B	110.0
O2^i^—Fe1—Cl1	95.48 (5)	H2A—C2—H2B	108.4
O2—Fe1—Cl1	90.85 (5)	N1—C3—C2^ii^	108.72 (15)
O1^i^—Fe1—Cl1	169.42 (4)	N1—C3—H3A	109.9
O1—Fe1—Cl1	91.53 (5)	C2^ii^—C3—H3A	109.9
O2^i^—Fe1—Cl1^i^	90.85 (5)	N1—C3—H3B	109.9
O2—Fe1—Cl1^i^	95.48 (5)	C2^ii^—C3—H3B	109.9
O1^i^—Fe1—Cl1^i^	91.53 (5)	H3A—C3—H3B	108.3
O1—Fe1—Cl1^i^	169.42 (4)	N1—C4—C4^ii^	108.77 (11)
Cl1—Fe1—Cl1^i^	98.47 (4)	N1—C4—H4A	109.9
C4—N1—C3	109.93 (18)	C4^ii^—C4—H4A	109.9
C4—N1—C2	109.49 (17)	N1—C4—H4B	109.9
C3—N1—C2	110.04 (18)	C4^ii^—C4—H4B	109.9
C4—N1—H1	109.1	H4A—C4—H4B	108.3
C3—N1—H1	109.1	C1—O1—Fe1	116.67 (12)
C2—N1—H1	109.1	Fe1—O2—H2WA	122.9 (15)
O3—C1—O1	126.47 (18)	Fe1—O2—H2WB	122 (2)
O3—C1—C1^i^	119.75 (12)	H2WA—O2—H2WB	115 (3)

Symmetry codes: (i) −*x*, *y*, −*z*+2; (ii) −*x*+1, *y*, −*z*+1.

### Hydrogen-bond geometry (Å, °)

**Table:**

*D*—H···*A*	*D*—H	H···*A*	*D*···*A*	*D*—H···*A*
N1—H1···O1^iii^	0.91	1.93	2.814 (2)	162
O2—H2WA···O3^iv^	0.87 (3)	1.86 (3)	2.722 (2)	168 (2)
O2—H2WB···Cl2	0.82 (3)	2.23 (3)	3.0359 (17)	170 (3)

Symmetry codes: (iii) *x*, *y*, *z*−1; (iv) −*x*+1/2, *y*+1/2, −*z*+2.

## References

[bb1] Flack, H. D. (1983). *Acta Cryst.* A**39**, 876–881.

[bb2] Fu, Y. L., Liu, Y. L., Shi, Z., Li, B. Z. & Pang, W. Q. (2002). *J. Solid State Chem.* **163**, 427–435.

[bb3] Keene, T. D., Hursthouse, M. B. & Price, D. J. (2004). *Acta Cryst.* E**60**, m378–m380.

[bb4] Lee, M. Y. & Wang, S. L. (1999). *Chem. Mater.* **11**, 3588–3594.

[bb5] Rigaku (2005). *CrystalClear.* Rigaku Corporation, Tokyo, Japan.

[bb6] Sheldrick, G. M. (2008). *Acta Cryst.* A**64**, 112–122. 10.1107/S0108767307043930 18156677

[bb7] Sukhendu, M. & Srinivasan, N. (2007). *Chem. Eur. J.* **13**, 968–977.

[bb8] Zhao, J. & Xu, L. (2008). *Inorg. Chim. Acta*, **361**, 2385–2395.

